# Protective Effect of Zuojin Fang on Lung Injury Induced by Sepsis through Downregulating the JAK1/STAT3 Signaling Pathway

**DOI:** 10.1155/2021/1419631

**Published:** 2021-01-06

**Authors:** Jiangning Yin, Zhan Yu, Chuanyong Hou, Yigen Peng, Jianpeng Xiao, Jun Jiang

**Affiliations:** ^1^Emergency Department, The Affiliated Jiangning Hospital of Nanjing Medical University, Nanjing, Jiangsu 211100, China; ^2^School of Pharmacy, Jiangsu University, Xuefu Road, Zhenjiang, Jiangsu 212013, China

## Abstract

Lung injury was the common and serious complication of sepsis, a systemic inflammatory response syndrome caused by severe infections. Chinese medicine had unique advantages in attenuating inflammatory response, such as Zuojinfang (ZJF). ZJF was a classical compound herb formula composed of Coptidis Rhizoma and Euodiae Fructus in a ratio of 6 : 1. In this paper, 15 ingredients in ZJF were identified and 8 of them absorbed into rat's serum were quantified by HPLC-MS/MS. Subsequently, sepsis-induced lung injury model was replicated in rats by cecal ligation and puncture. 60 SD rats were randomly divided into 6 groups (*n* = 10): control group (CON), sham group (Sham), model group (MOD), ZJF low-dose group (ZJF-L), ZJF high-dose group (ZJF-H), and prednisolone group (PNSL). Within the next 24 h, the levels of inflammatory factors, correlation between active ingredients and inflammatory cytokines, the pathological changes of lung tissue, and protein expression of the JAK1/STAT3 signaling pathways were analyzed one by one. Finally, the concentration order of components absorbed in rat serum was berberine > palmatine > jatrorrhizine > coptisine > evodin > chlorogenic acid > evodiamine. Compared with the MOD group, the TNF-*α*, IL-6, and IFN-*γ* in the ZJF-H group were significantly reduced (*p* < 0.05). Moreover, the TNF-*α* decreased significantly accompanied by the increase of berberine, chlorogenic acid, jatrorrhizine, palmatine, evodin, and evodiamine in serum (negative correlation, *p* < 0.05). Compared with the MOD, the area of lung injury, the expressions of JAK1, p-JAK1, STAT3, and p-STAT3 were significantly decreased under the treatment of ZJF (*p* < 0.05). Therefore, downregulating the JAK1/STAT3 signaling pathways was a potential avenue of ZJF in reversing lung injury induced by sepsis.

## 1. Introduction

Sepsis was defined as a life-threatening organ dysfunction caused by a dysregulated host response to infection [1]. At present, sepsis was a leading cause of death and disability in intensive care units (ICU). The incidence of sepsis was between 66 and 300 per 100000 people and showed an increasing trend [2–4]. Mortality estimates for sepsis ranged from 27 to 36% in developed countries [5]. In its preliminary stage, sepsis tended to induce lung injury, characterized by damaged alveolar capillary barrier, edema, pulmonary infiltrates, and hypoxemia [6]. Nowadays, treatment of sepsis-induced lung injury includes antibiotic [7], glucocorticoids [8], corticosteroid [9], ketamine [10], simvastatin [11], and artesunate [12]. However, the use of antibiotics inevitably leads to the emergence of resistant bacteria, a series of adverse reactions [13, 14]. Other drugs were also accompanied by some troubling side effects in osteoporosis and glaucoma [15], psychiatric adverse effect [16], cardiovascular diseases [17], liver and muscle injury [18], and cytotoxicity [19], respectively.

Fortunately, it was found that some Chinese herbs had unique advantages in reducing the production of drug-resistant bacteria and inhibiting the generation of proinflammatory factors. Moreover, active ingredients obtained from Chinese herbs or compound preparation of Chinese medicine like emodin [20], ginsenoside Rg_1_ [21], glycyrrhizic acid [22], and Xuebijing injection [23] usually provided beneficial effects in anti-infective, anti-inflammatory, and immunomodulatory.

Zuojinfang (ZJF) was a classical formula composed of Coptidis Rhizoma (CR) and Euodiae Fructus (EF) in a ratio of 6 : 1. ZJF was always applied to treat gastric ulcer, gastroesophageal reflux, gastritis, and pyloric obstruction [24, 25]. The active ingredients of CR are alkaloids, including berberine, jatrorrhizine, palmatine, and coptisine; especially, berberine is the most abundant compound [26]. Studies showed that CR extracts and berberine can effectively improve the survival rate of rats with sepsis [27]. Furthermore, Huang-Lian-Jie-Du Decoction has been widely used in the treatment of sepsis by activating the choline pathway and inhibiting the HMGB-1/TLR4/NF-*κ*B signaling pathway [28]. In addition, relevant studies have proved that evodiamine has a therapeutic effect on inflammation by reducing the expression of IL-1*β*, IL-6, and TNF-*α* [29]. Based on these researches, it was believed that ZJF has a unique pharmacological action in reversing sepsis-induced lung injury.

The purpose of this study was to verify the efficacy and mechanism of ZJF in reversing lung injury induced by sepsis. Firstly, HPLC-MS/MS was applied for the qualitative and quantitative analysis of ZJF. And then the 8 ingredients absorbed into the rat's serum were also determined by HPLC-MS/MS. The inflammatory factor levels were tested by enzyme-linked immunosorbent assay (ELISA). Pathologic changes in lung tissues were evaluated via pathological section with hematoxylin and eosin (HE) staining. The expressions of JAK, p-JAK1, STAT3, and p-STAT3 were determined by western blot assay. Our study not only evaluated the therapeutic effect of ZJF on lung injury induced by sepsis but also provided a new perspective for resolving the mechanism of ZJF in regulating the JAK1/STAT3 signaling pathway.

## 2. Materials and Method

### 2.1. Ethics Statement

Animal experiments were carried out in accordance with the Guidelines of Animal Experimentation in Jiangsu University (Zhenjiang, China), and the protocol was also approved by its Animal Ethics Committee.

### 2.2. Reference Materials and Medicinal Materials

Chlorogenic acid, jatrorrhizine, palmatine, berberine, evodin, evodiamine, and rutaecarpine (purity > 98.0%) were purchased from the National Institute for the Control of Pharmaceutical and Biological Products (Beijing, China). Coptisine (purity > 98.5%) was purchased from Prof. Hao Zhang (West China School of Pharmacy, Sichuan University, Chengdu, China). CR was provided by Nanjing Pharmacy (Nanjing, China). EF was provided by Evodiae Planting Base (Hubei, China). CR and EF were identified by Prof. Chengying Wu (Jiangsu Provincial Academy of Chinese Medicine, Nanjing, China) as *Coptidis Rhizoma Franch* and *Evodiarutaecar pa (Juss.) Benth.* var. officinalis (Dode) Huang, respectively. ZJF was obtained by mixing CR (120 g) and EF (20 g), crushing, and passing through 60 molybdenum sieves.

### 2.3. Model Establishment and Grouping

Cecal ligation and puncture [30–33] was applied to establish sepsis model in rats. 60 healthy male SD rats (SPF, 220 ± 20 g) were provided by the Animal Center of Jiangsu University. All rats were randomly divided into 6 groups (*n* = 10): control group (CON, normal saline, 10 mL/kg), sham operation group (Sham, normal saline, 10 mL/kg), model group (MOD, normal saline, 10 mL/kg), ZJF low-dose group (ZJF-L, 20 mg/kg/day), ZJF high-dose group (ZJF-H, 40 mg/kg/day), and positive control prednisolone group (PNSL, 10 mg/kg/day).

### 2.4. Preparation of Standard and Sample Solutions

#### 2.4.1. Standard Solutions

Mixed standard stock solution containing chlorogenic acid (34.5 *μ*g/mL), jatrorrhizine (35.0 *μ*g/mL), coptisine (18.0 *μ*g/mL), palmatine (13.0 *μ*g/mL), berberine (43.0 *μ*g/mL), evodin (35.0 *μ*g/mL), evodiamine (42.0 *μ*g/mL), and rutaecarpine (32.0 *μ*g/mL) was prepared in methanol. The solutions were stored in refrigerator at 4°C and kept in darkness. The mixed standard stock solution was diluted into a series of standard working solution by methanol.

#### 2.4.2. Preparation of Extract Samples

ZJF (CR 120 g and EF 20 g) was extracted twice with 65% ethanol by refluxing for 90 min each time [34]. Two-part extraction was incorporated, evaporated under vacuum, and lyophilized to obtain the ZJF extracts. Freeze-dried powder (0.1 g) was weighed into a 50 mL polypropylene centrifugal tube, and 50 mL of methanol was added for sonication about 40 min. Before analysis by HPLC, the supernatant solution was filtered through a 0.45 *μ*m pore size Millipore filter, and then a total volume of 10 *μ*L was injected.

#### 2.4.3. Preparation of Serum Samples

After intragastric administration of ZJF extracts (freeze-dried powder dispersed in 0.5% CMC-Na), the rats were subjected to blood collection from orbit at 6 h, 12 h, and 24 h, respectively. 0.2 mL of plasma was taken into the heparinized centrifuge tube, centrifuged at 4000 rpm for 10 min, and 50 *μ*L of supernatant was taken and frozen-spare.

0.5 mL of ethyl acetate was added to 50 *μ*L of the serum, vortexed for 2 min, and centrifuged for 10 min to obtain 400 *μ*L of the supernatant. It was vacuum-dried at 40°C until ethyl acetate was evaporated, reconstituted by adding 100 *μ*L of methanol, ultrasonically mixed for 5 min, and centrifuged for 10 min to obtain 80 *μ*L of the supernatant for chromatographic analysis.

### 2.5. Chromatographic and Mass Spectrometry Conditions

ZORBAX XDC-C_18_ column (4.6 mm × 50 mm, 1.8 *μ*m) was used for the chromatographic separation under gradient elution with acetonitrile -0.3% glacial acetic acid (HCN%, 0-7 min: 5-8; 7-13 min: 8-11 min; 13-25 min: 11-25; 25-45 min: 25-32; 45-50 min: 30; 50-75 min: 30-60; flow rate: 0.4 mL/min) at a flow rate 0.4 mL/min. The column injection volume was 5 *μ*L, and column temperature was 20°C. MS acquisition was carried out in the ESI-positive ionization mode. Detection mode was set to multiple reaction monitoring (MRM, Table 1). The conditions were set as follows: corona discharge current 1.16 *μ*A, capillary voltage 4000 V, a nitrogen flow rate 10 L/min, gas temperature 350°C, scan range 100-600 (m/z), and nebulizer pressure 25 psi.

### 2.6. Method Validation of HPLC-MS/MS

#### 2.6.1. Regression Equations, Linear Range, and LOD of 8 Components

**Figure 1:**
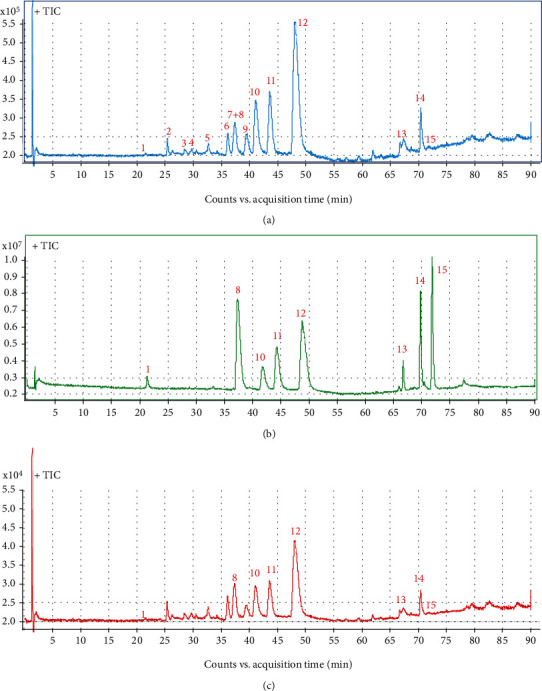
Typical total ion chromatogram (TIC) of 15 active ingredients in ZJF extracts. (a) ZJF sample. (b) Standard solution. (c) Serum sample. Peaks: 1: chlorogenic acid; 8: jatrorrhizine; 10: coptisine; 11: palmatine; 12: berberine; 13: evodin; 14: evodiamine; 15: rutaecarpine.

The stock solutions of eight standards were prepared and diluted to six appropriate concentrations for establishing the calibration curves of HPLC-MS/MS (Figure 1). The regression equations were achieved after linear regression of the peak areas versus the corresponding concentrations. The LOQ for each analyte under the chromatographic conditions was determined at the S/N of 3 and 10, respectively. The results indicated that all eight reference compounds had a good linearity (*r*^2^ > 0.9997) in a relatively wide concentration range (Table 2).

#### 2.6.2. Precision, Repeatability, Stability, and Recovery

Intraday and interday variations were chosen to determine the precision of the developed assay. Three different concentrations of standards were prepared. The intraday variation was determined by analyzing the six replicates within a single day. Interday variation was examined in six consecutive days. Repeatability was confirmed with solutions prepared from ZJF, and it was injected into the apparatus at 0, 2, 4, 8, 12, 16, and 24 h, respectively. Variations were expressed by RSD. It indicated that the intraday, interday, repeatability, and stability of RSD values of the eight compounds were all less than 3.0%.

10 *μ*L mixed standard solution (10 ppm) was precisely removed into 0.1 mL blank plasma samples and their peak areas were recorded (*A*) by HPLC-MS/MS after the same pretreatment as described in Section 2.4. Another 10 *μ*L mixed standard solution (10 ppm) was added into 0.1 mL methanol with the same operation and their areas were measured as “*B*.” Their recovery was calculated by “(*A*/*B*) × 100%”. The recovery rate of 8 active ingredients in serum showed stable recovery between 83.7% and 89.5%.

### 2.7. Detection of TNF-*α*, IFN-*γ*, IL-6, and SOD in Serum

Detection was performed according to the manufacturer's instructions for the enzyme-linked immunosorbent assay kits. 50 *μ*L standard solution of series concentrations and 10 *μ*L sample solution were prepared; then, the diluent was added (40 *μ*L). Standard and sample wells were added with horseradish peroxidase- (HRP-) labelled antibody (100 *μ*L) to detect the antibody. The reaction wells were sealed with the sealing plate membrane and kept in 37°C water bath for 60 min. Discard the liquid, dry the plates, fill them up with washing fluid for 1 min, shake off the washing liquid, dry the plates, and repeat the washing process 5 times. The substrates were added (50 *μ*L) to all the wells and incubated for 15 min at 37°C. 15 min later, the OD value of each well was measured at 450 nm wavelength. The standard curve was obtained with the linear regression of standard concentration and OD value, and the corresponding concentration of the OD value of the sample was found on this curve. The concentration of samples could be found from the standard curve according to their OD values.

### 2.8. HE Staining

Collected lung tissues were fixed in 10% formalin for 48 h. The right upper lobe and hilar organizations were routinely washed, dehydrated, embedded in paraffin, and cut into 5 *μ*m serial sections. Then, the sections were gradually dewaxed, dehydrated, stained with HE, immersed in xylene and alcohol, stained with hematoxylin for 5 min, stained with eosin for 3 min, and reimmersed in alcohol and xylene. Finally, the morphological changes [35, 36] of tissues were observed under an optical microscope (Olympus IX71/IX81, Olympus Corporation, Japan).

### 2.9. Expression of JAK1, p-JAK1, STAT3, and p-STAT3 in Lung Tissue

Protein samples of lung tissue were prepared and subjected to protein gel electrophoresis (Bio-Rad). The gel was transferred to a membrane, blocked, and incubated with a primary antibody: the JAK1 (Santa Cruz, CA, USA; sc-376996), p-JAK1 (Santa Cruz, CA, USA; sc-377043), STAT3 (Santa Cruz, CA, USA; sc-482), and p-STAT3 (Santa Cruz, CA, USA; sc-8001-R) diluted 1 : 1000. The diluted primary antibody was incubated at room temperature for 2 h. Secondary antibody was incubated for 30-60 min. Membranes were visualized with enhanced chemiluminescence. ImageJ software (National Institutes of Health, Bethesda, MD) was used for analyzing protein bands. The relative density of each band was calculated to *β*-actin as the control.

### 2.10. Statistical Analysis

All data were presented as the mean ± SD. Statistical analysis was performed using GraphPad Prism 5 (GraphPad software, USA). Differences were analyzed by one-way analysis of variance (Tukey/compare all pairs of columns). Differences *p* < 0.05 were considered as significant.

## 3. Results

### 3.1. Profiling Chemical Composition in ZJF

**Figure 2:**
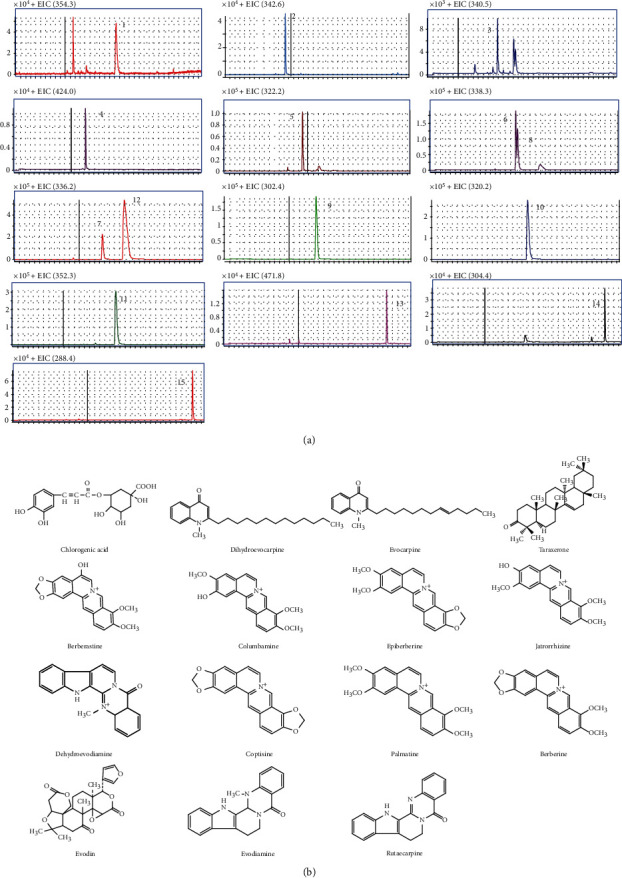
Qualitative analysis of active components in ZJF. (a) Extracting ion chromatogram (EIC) of 15 components in ZJF extract. (b) Chemical structures of identified 15 components in ZJF extract.

The extracted ion chromatogram (EIC) of 15 components (Figure 2) was retrieved in ZJF according to retention time (*t*_R_) and corresponding parent ion (m/z) produced by HPLC-MS/MS (Table 1). They were chlorogenic acid (1), dehydroevodiamine (2), evocarpine (3), taraxerone (4), berberastine (5), columbamine (6), epiberberine (7), jatrorrhizine (8), dehydroevodiamine (9), coptisine (10), palmatine (11), berberine (12), evodin (13), evodiamine (14), and rutaecarpine (15). Based on the external standard method, the contents of compounds 1, 8, and 10-15 were 0.64 ± 0.14 mg/g, 13.15 ± 2.64 mg/g, 38.16 ± 4.51 mg/g, 14.97 ± 2.89 mg/g, 79.06 ± 8.35 mg/g, 2.52 ± 0.56 mg/g, 0.56 ± 0.12 mg/g, and 0.35 ± 0.08 mg/g, respectively. The content of berberine was the highest and rutaecarpine was the lowest in ZJF extracts (Supplementary Materials).

### 3.2. Dynamic Changes of Ingredients Absorbed into Serum

The contents of the above 8 components in serum at 6 h, 12 h, and 24 h after oral administration of ZJF at high and low doses were quantitatively analyzed. Finally, only the rutaecarpine was not detected, and the other 7 components were successfully quantified. Results showed that the content of Coptis alkaloids in serum was always the highest, including berberine (100.28 ± 2.31 ng/mL), jatrorrhizine (11.42 ± 0.71 ng/mL), coptisine (9.33 ± 0.43 ng/mL), and palmatine (20.73 ± 1.55 ng/mL), especially the berberine. Besides, the contents of evodin (2.64 ± 0.44 ng/mL) and chlorogenic acid (3.85 ± 0.78 ng/mL) obtained from Euodiae Fructus were also prominent in serum. Furthermore, the content of these active ingredients in serum decreased significantly from 6 h to 24 h after administration (Table 3).

### 3.3. Dynamic Changes of Inflammation-Related Factors

**Figure 3:**
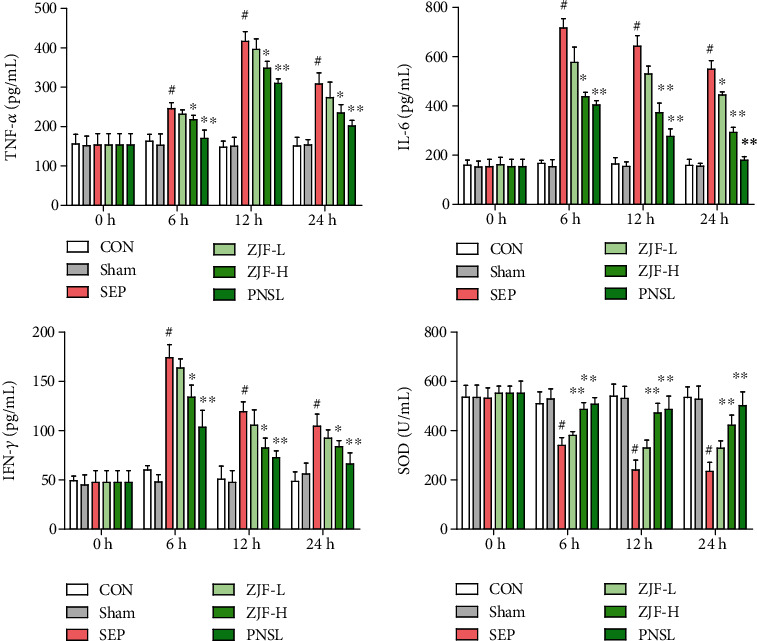
Dynamic changes of inflammation-related factors in rat serum. CON: control group; Sham: sham operation group; SEP: model group; ZJF-L: Zuojinfang low-dose group; ZJF-H: Zuojinfang high-dose group; PNSL: prednisolone; TNF-*α*: tumor necrosis factor *α*; IFN-*γ*: interferon *γ*; IL-6: interleukin 6; SOD: superoxide dismutase. ^∗^*p* < 0.05 and ^∗∗^*p* < 0.01 compared with the SEP group. ^#^*p* < 0.05 compared with the sham group.

TNF-*α*, IFN-*γ*, and IL-6 were the inflammatory factors, and SOD was an antioxidant index, which were related to inflammation. Compared with the sham group, the levels of TNF-*α*, IFN-*γ*, and IL-6 at 6 h, 12 h, and 24 h in the MOD group were all significantly increased (*p* < 0.05) along with the significant decrease of SOD (*p* < 0.05). After the intervention of ZJF and PNSL, the expressions of TNF-*α*, IFN-*γ*, and IL-6 were reduced and the SOD level was increased (Figure 3). More specifically, compared with the MOD group, the expressions of TNF-*α*, IL-6, and IFN-*γ* in ZJF-H group were significantly reduced (*p* < 0.05) and the level of SOD was significantly increased (*p* < 0.01). Therefore, ZJF significantly inhibited the inflammatory expression in septic rats.

### 3.4. Correlation Analysis

**Figure 4:**
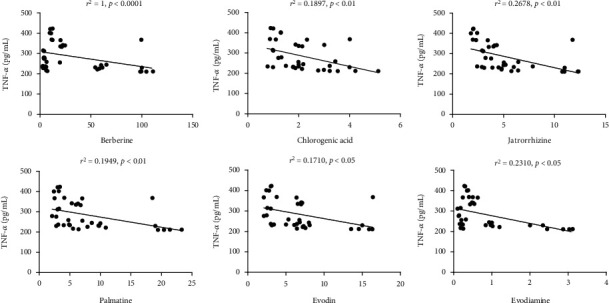
Correlation analysis between active components and the inflammatory factors in serum. There was a negative correlation between 6 active components (chlorogenic acid, jatrorrhizine, palmatine, berberine, evodiamine, and rutaecarpine) and TNF-*α* (*p* < 0.05).

The correlation analysis was conducted between the components and the inflammatory factors (TNF-*α*, IL-6, and IFN-*γ*) at same time points (6 h, 12 h, and 24 h). The levels of inflammatory cytokines were regarded as the ordinate, and the contents of active ingredients were the abscissa for linear regression (Figure 4). The results showed that the TNF-*α* was negatively correlated with berberine (*p* < 0.0001), chlorogenic acid (*p* < 0.01), jatrorrhizine (*p* < 0.01), palmatine (*p* < 0.01), evodin (*p* < 0.05), and evodiamine (*p* < 0.05). Moreover, there was no significant correlation between the other inflammatory factors (IL-6 and IFN-*γ*) and the active ingredients.

### 3.5. Pathological Changes in Lung Tissue

**Figure 5:**
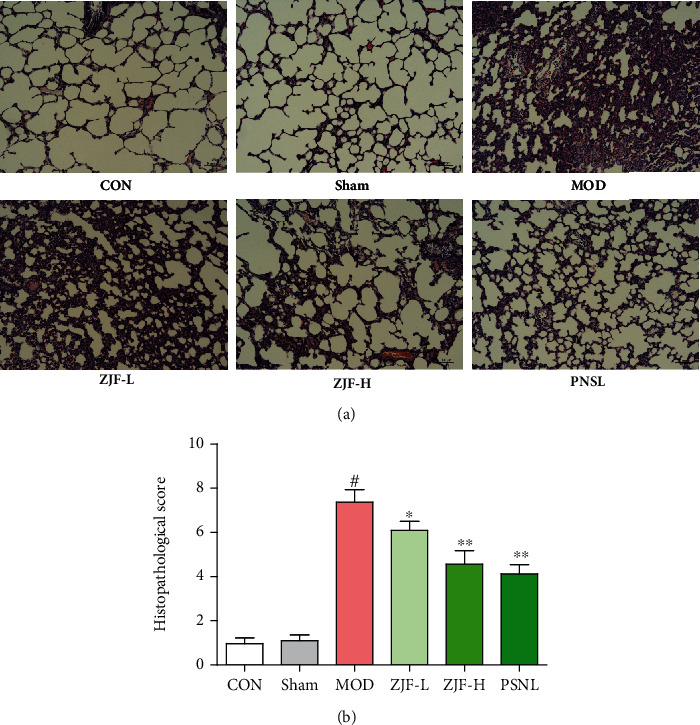
HE staining of lung pathological changes in septic rats. Stained sections were observed under optical microscope (×100). The lung injury scores were as follows: 0 points: normal kidney; 1 point: minimal damage (<10% lesion area); 2 points: mild damage (10%-30% lesion area); 3 points: moderate damage (30%-75% lesion area); 4 points: severe damage (>75% lesion area). Note. ^#^Compared with normal group, *p* < 0.01. ^∗∗^Compared with the model group, *p* < 0.01. ^∗^Compared with the model group, *p* < 0.05.

In the CON or Sham groups, the alveolar wall was thin; no exudate was found in the alveolar cavity and interstitium under the microscope. However, in the MOD group, dark red plaques or spot-like lesions of different sizes were observed on the lung surface accompanied by swelling and hyperemia of lung tissue. In the ZJF-L group, alveolar interstitium was hyperemia and edema, neutrophils were moderately infiltrated, capillaries were dilated, and some alveolar spaces were widened. In the ZJF-H group, alveolar interstitium was slightly congested and edematous with mild neutrophil infiltration, telangiectasia, and hyperemia (Figure 5(a)). Pathological score showed that the damage area in the MOD group was significantly increased compared with the sham group (*p* < 0.01). After treatment with ZJF and PNSL, the area of lung injury decreased significantly compared with the MOD (*p* < 0.05, Figure 5(b)).

### 3.6. Expression of JAK1, p-JAK1, STAT3, and p-STAT3 in Lung Tissue

**Figure 6:**
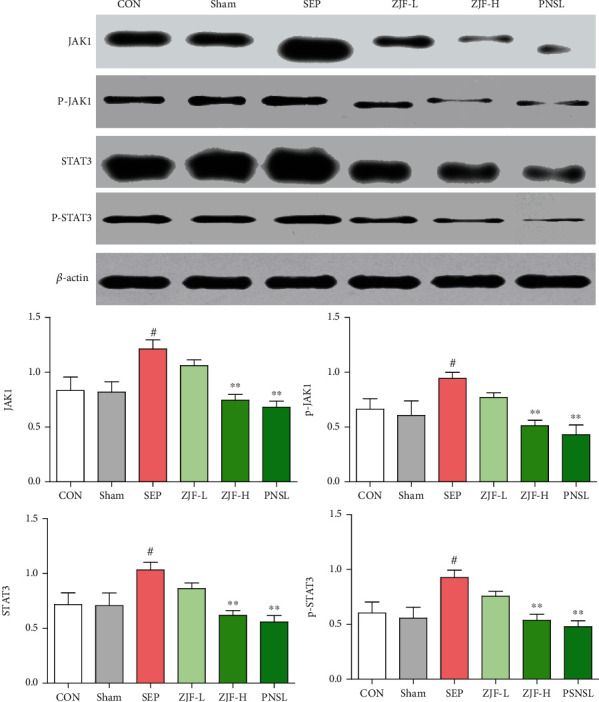
Expression of JAK1, p-JAK1, STAT3, and p-STAT3 proteins in rat lung tissue. CON: control group; Sham: sham operation group; SEP: model group; ZJF-L: Zuojinfang low-dose group; ZJF-H: Zuojinfang high-dose group; PNSL: prednisolone; JAK1: Janus kinase 1; STAT3: signal transducer and activator of transcription. ^∗∗^*p* < 0.01 compared with the SEP group. ^#^*p* < 0.05 compared with the sham group.

Compared with the sham group, the protein expressions of JAK1, p-JAK1, STAT3, and p-STAT3 in the MOD were increased (*p* < 0.05). Compared with the MOD, the PNSL also significantly downregulated the protein expression of JAK1, p-JAK1, STAT3, and p-STAT3 (*p* < 0.01). Similar to the PNSL group, the expression of JAK1, p-JAK1, STAT3, and p-STAT3 was also downregulated in the ZJF-H group (*p* < 0.01). Therefore, ZJF reversed sepsis caused lung injury by downregulating the expression of JAK1/STAT3-related proteins (Figure 6).

## 4. Discussion

According to our previous studies, the contents of the 8 components in ZJF extract were in the order of berberine > coptisine > palmatine > jatrorrhizine > evodin > chlorogenic acid > evodiamine > rutaecarpine. After oral administration of ZJF, the concentration of components absorbed into the blood is in this order berberine > palmatine > jatrorrhizine > coptisine > evodin > chlorogenic acid > evodiamine. Therefore, the contents of berberine, palmatine, jatrorrhizine, coptisine, evodin, chlorogenic acid, and evodiamine in both the extract and the serum were prominent. However, in Chinese Pharmacopoeia, berberine was also utilized as the only indicator for the quality control of ZJF. Simultaneous determination of multicomponents established in this study provided an important technical support for the quality control of ZJF and the subsequent investigation of integrative mechanism.

Undoubtedly, excessive inflammation response was the major contributing factor for sepsis-induced organ injury [37]. This immune dysregulation was a complex interplay of diverse signaling pathways which were aberrantly activated or suppressed losing their normal regulated functions. Excessive expression of inflammatory mediators in sepsis was associated with multiple signaling pathways, such as Janus kinase (JAK)/signal transducer and activator of transcription (STAT). The JAK/STAT signaling pathway leads to the accumulation of a large number of proinflammatory cytokines and increases the inflammatory response. In recent years, the significance of JAK1/STAT3 signaling involved in regulating inflammation and injury of sepsis attracted more attention due to its simple and effective activation. Sepsis induced JAK1 phosphorylation, followed by STAT3 phosphorylation, which activated the JAK1/STAT3 signaling pathway. Subsequently, the inflammatory response was increased under the trigger of p-STAT3, thereby increasing the severity of lung injury [38]. It was showed that the continued deterioration of sepsis is associated with excessive expression of inflammatory factors caused by the JAK1-STAT3 signaling pathway. Similarly, when the JAK1-STAT3 signaling pathway was inhibited, it reduced the severe inflammatory response caused by sepsis. After oral administration of ZJF, the expressions of JAK1, p-JAK1, STAT3, and p-STAT3 proteins were significantly reduced (*p* < 0.05) and ultimately reduced the level of inflammatory mediators (*p* < 0.05). Therefore, this study confirmed that ZJF could reverse sepsis-induced lung injury by inhibiting the JAK1-STAT3 signal pathway and reducing the expression of inflammatory factors.

In order to explore the intrinsic communication of components and inflammation, the correlations between 8 components and 3 inflammatory factors (TNF-*α*, IFN-*γ*, and IL-6) in serum were analyzed according to the data collected at 6 h, 12 h, and 24 h after oral administration of ZJF extract. Fortunately, the concentration of TNF-*α* decreased significantly accompanied by the increase of various components, including berberine (*p* < 0.0001), chlorogenic acid (*p* < 0.01), jatrorrhizine (*p* < 0.01), palmatine (*p* < 0.01), evodin (*p* < 0.05), and evodiamine (*p* < 0.05). However, this notable negative correlation did not exist in other inflammatory factors and ingredients. This study suggested that berberine, chlorogenic acid, jarrorhizine, palmatine, evodin, and evodiamine had inhibitory effects on sepsis-induced inflammatory response, especially the TNF-*α*. Through literature review, other laboratory also found that berberine [39] and chlorogenic acid [40, 41] could inhibit the expression of inflammatory factors through the JAK/STAT signaling pathway.

There are few studies on antisepsis of ZJF, Coptis chinensis (CR), Evodia rutaecarpa (EF), and their main components (berberine and rutaecarpine). In the existing reports, it was found that ZJF, CR, and EF downregulated the expression of inflammatory factors, such as TNF-*α*. The main mechanism was related to the NF-*κ*B signaling pathway [42, 43]. However, the expression of inflammatory cytokines (TNF-*α*, IL-6, IL-1*β*, and so on) can also be regulated through other signaling pathways, such as JAK-STAT [44–46]. Therefore, the goal of this paper was to prove that ZJF, CR, and EF exerted antisepsis effect by inhibiting the JAK-STAT signaling pathway to downregulate the expression of inflammatory factors. Fortunately, it was found that there was a negative correlation between serum concentrations of multiple components and TNF-*α* levels. Therefore, downregulating the level of inflammatory factors by inhibiting the JAK-STAT signaling pathway was one of the mechanisms of ZJF for the treatment of sepsis. ZJF reversed the lung injury induced by sepsis was related to the inhibition of inflammatory response under the synergistic effect of multicomponents, such as chlorogenic acid, jatrorrhizine, palmatine, berberine, evodiamine, and rutaecarpine. In the subsequent study, the mechanism of these components in treating lung injury induced by sepsis will be continued to explore.

## 5. Conclusions

ZJF reversed the lung injury caused by sepsis through inhibiting JAK1/STAT3 expression and subsequently reducing inflammatory mediator production. More importantly, berberine, chlorogenic acid, jarrorhizine, palmatine, evodin, and evodiamine were important potential substances of ZJF in treating lung injury induced by sepsis.

## Figures and Tables

**Table 1:** Screening parameters of the compounds in ZJF by HPLC-MS/MS.

Compounds	*t*_R_^a^ (min)	m/z [M]^+^	m/z [M + H]^+^
1	20.87		355.3
2	24.60		342.6
3	27.74		340.5
4	28.76	424.0	
5	31.56	322.2^b^	
6	34.27	338.3	
7	34.95	336.2	
8	35.12	338.3	
9	36.90	302.4	
10	38.18	320.2	
11	40.47	352.3	
12	44.71	336.2	
13	65.66	471.8	
14	68.55		304.4
15	70.58		288.4

^a^Times of retention. ^b^[M-HCHO]^+^.

**Table 2:** Regression equations, correlation coefficient, linear range, LOD, and recovery of 8 components by HPLC-MS/MS.

Compounds	Regression equation	*r* ^2^	Linear range (ng/mL)	LOQ (ng/mL)	Recovery (%)
1	*Y* = 5053.5*X* − 282.5	0.9999	0.5-50.0	0.5	85.4
8	*Y* = 1048721*X* + 101447	0.9999	0.5-50.0	0.5	87.2
10	*Y* = 258201*X* − 40.1	0.9999	0.5-50.0	0.5	83.7
11	*Y* = 508402*X* − 28108	0.9999	0.5-50.0	0.5	88.1
12	*Y* = 1271407*X* − 111917	0.9997	2.0-150.0	2.0	86.8
13	*Y* = 32151*X* − 3568.5	0.9999	0.5-50.0	0.5	86.3
14	*Y* = 211093*X* − 688.8	0.9998	0.1-10.0	0.1	88.2
15	*Y* = 838756*X* − 77674	0.9998	0.1-10.0	0.1	89.5

Note: *Y* is the peak area; *X* is the concentration injected. LOQ refers to the limit of quantification. 1: chlorogenic acid; 8: jatrorrhizine; 10: coptisine; 11: palmatine; 12: berberine; 13: evodin; 14: evodiamine; 15: rutaecarpine.

**Table 3:** Concentration changes of ZJF active ingredients in rat serum after different treatment time (*n* = 3).

Dose	*P*_t_	Contents (ng/mL)							
		Chlorogenic acid	Jatrorrhizine	Coptisine	Palmatine	Berberine	Evodin	Evodiamine	Rutaecarpine
ZJF-L (20 mg/kg)	6 h	1.96 ± 0.17	4.70 ± 0.46	3.75 ± 0.63	9.65 ± 0.82	59.95 ± 3.10	7.25 ± 0.75	0.99 ± 0.12	nd
	12 h	1.08 ± 0.19	2.09 ± 0.22	3.15 ± 0.37	3.10 ± 0.62	11.09 ± 1.17	2.86 ± 0.58	0.33 ± 0.06	nd
	24 h	1.13 ± 0.26	3.03 ± 0.46	1.78 ± 0.53	3.04 ± 0.62	3.87 ± 0.77	2.76 ± 0.47	0.15 ± 0.04	nd
ZJF-H (40 mg/kg)	6 h	3.85 ± 0.78	11.42 ± 0.71	9.33 ± 0.43	20.73 ± 1.55	100.28 ± 2.31	14.75 ± 1.26	2.64 ± 0.44	nd
	12 h	2.56 ± 0.81	4.04 ± 0.50	7.64 ± 0.60	6.50 ± 0.79	21.32 ± 1.89	6.74 ± 0.33	0.52 ± 0.07	nd
	24 h	2.74 ± 0.62	6.26 ± 0.95	3.97 ± 0.41	5.62 ± 0.94	6.30 ± 0.60	6.25 ± 0.94	0.23 ± 0.05	nd

## Data Availability

The data used to support the findings of this study are available from the corresponding author upon request.

## Supplementary Materials

Supplementary Materials Table 1: content of 8 active components in ZJF extract (mg/g, *n* = 3). Figure 1: typical HPLC-DAD chromatograms of the standard solution and ZJW extract.
