# Supplementary material for: Protective Effect of Zuojin Fang on Lung Injury Induced by Sepsis through Downregulating the JAK1/STAT3 Signaling Pathway
Source: Biomed Res Int. 2021 Jan 6;2021:1419631. doi: 10.1155/2021/1419631 (PMC7808815; doi:10.1155/2021/1419631)

**Table 1S.** Content of 8 active components in ZJF extract (mg/g, n=3).

| Contents of analytes (mg/g, n = 3) | | | | | | | |
| --- | --- | --- | --- | --- | --- | --- | --- |
| 1 | 2 | 3 | 4 | 5 | 6 | 7 | 8 |
| 0.66 | 13.68 | 38.91 | 15.04 | 81.74 | 2.43 | 0.59 | 0.39 |
| 0.56 | 11.43 | 42.55 | 13.43 | 78.33 | 2.13 | 0.47 | 0.26 |
| 0.63 | 11.76 | 38.93 | 11.58 | 69.64 | 1.43 | 0.63 | 0.42 |
| 0.48 | 13.87 | 34.23 | 17.88 | 64.47 | 2.76 | 0.61 | 0.39 |
| 0.58 | 13.93 | 32.54 | 16.15 | 68.42 | 2.26 | 0.53 | 0.40 |
| 0.61 | 10.63 | 31.43 | 16.81 | 72.37 | 2.19 | 0.55 | 0.52 |
| 0.55 | 9.54 | 29.01 | 17.76 | 86.31 | 3.32 | 0.67 | 0.33 |
| 0.44 | 15.43 | 36.91 | 12.15 | 90.26 | 2.54 | 0.49 | 0.35 |
| 0.73 | 12.51 | 34.80 | 14.81 | 74.21 | 2.67 | 0.41 | 0.46 |
| 0.92 | 10.50 | 42.70 | 19.48 | 88.16 | 2.40 | 0.53 | 0.38 |
| 0.81 | 15.49 | 40.59 | 12.15 | 72.11 | 1.73 | 0.45 | 0.29 |
| 0.40 | 14.49 | 38.49 | 10.81 | 96.06 | 2.44 | 0.68 | 0.36 |
| 0.59 | 17.48 | 46.38 | 12.28 | 88.58 | 3.48 | 0.80 | 0.27 |
| 0.68 | 14.48 | 34.28 | 11.74 | 80.03 | 1.73 | 0.52 | 0.23 |
| 0.77 | 16.47 | 42.17 | 17.21 | 71.47 | 3.18 | 0.48 | 0.39 |
| 0.76 | 18.46 | 40.07 | 16.68 | 82.92 | 2.06 | 0.49 | 0.35 |
| 0.45 | 11.46 | 37.96 | 18.14 | 74.37 | 2.94 | 0.88 | 0.25 |
| 0.64 | 10.45 | 35.86 | 19.61 | 85.82 | 2.82 | 0.60 | 0.37 |
| 0.73 | 11.45 | 43.75 | 14.08 | 77.27 | 2.69 | 0.39 | 0.24 |
| 0.82 | 9.44 | 41.65 | 11.54 | 78.72 | 3.27 | 0.49 | 0.35 |

1. Chlorogenic acid, 2. Jatrorrhizine, 3. Coptisine, 4. Palmatin, 5. Berberine, 6. Evodin, 7. Evodiamine, 8. Rutaecarpine.

**Legends to Figures**

**Figure 1S. Typical HPLC-DAD chromatograms of the standard solution and ZJW extract.**

A. Standard solution, B. ZJW. Peaks: 1. Chlorogenic acid, 2. Jatrorrhizine, 3. Coptisine, 4. Palmatin, 5. Berberine, 6. Evodin, 7. Evodiamine, 8. Rutaecarpine

**Figure 1S**


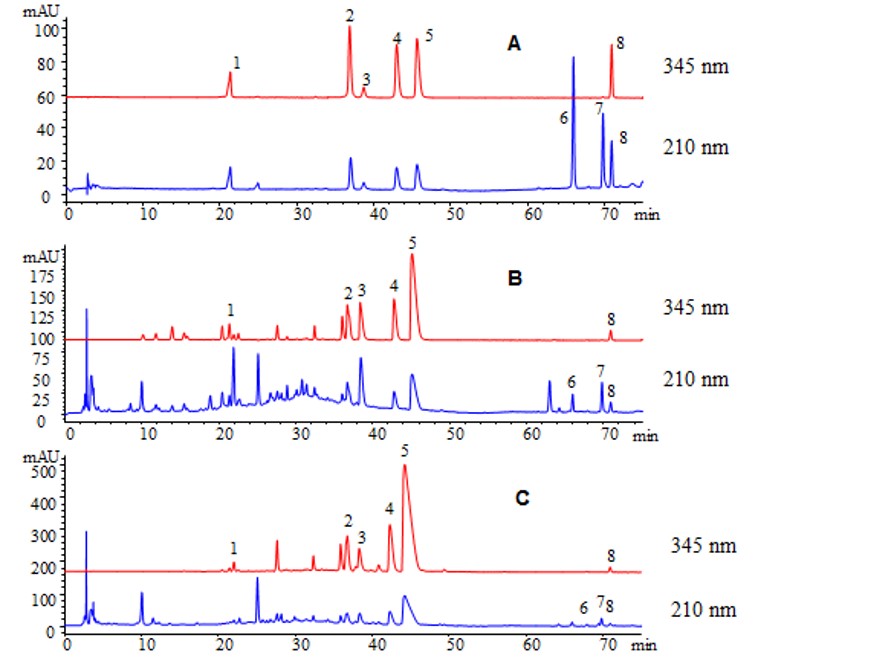

Supplement: Supplementary Materials — Table 1: content of 8 active components in ZJF extract (mg/g, n = 3). Figure 1: typical HPLC-DAD chromatograms of the standard solution and ZJW extract. [file 1419631.f1.docx]
